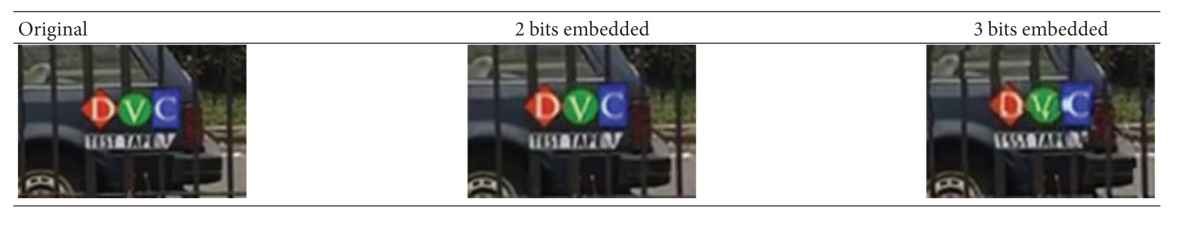# A Robust H.264/AVC Video Watermarking Scheme with Drift Compensation

**DOI:** 10.1155/2014/802347

**Published:** 2014-02-04

**Authors:** Xinghao Jiang, Tanfeng Sun, Yue Zhou, Wan Wang, Yun-Qing Shi

**Affiliations:** ^1^School of Electronic Information and Electrical Engineering, Shanghai Jiao Tong University, Shanghai 200240, China; ^2^Department of Electrical and Computer Engineering, New Jersey Institute of Technology, Newark, NJ 07102, USA

## Abstract

A robust H.264/AVC video watermarking scheme for copyright protection with self-adaptive drift compensation is proposed. In our scheme, motion vector residuals of macroblocks with the smallest partition size are selected to hide copyright information in order to hold visual impact and distortion drift to a minimum. Drift compensation is also implemented to reduce the influence of watermark to the most extent. Besides, discrete cosine transform (DCT) with energy compact property is applied to the motion vector residual group, which can ensure robustness against intentional attacks. According to the experimental results, this scheme gains excellent imperceptibility and low bit-rate increase. Malicious attacks with different quantization parameters (QPs) or motion estimation algorithms can be resisted efficiently, with 80% accuracy on average after lossy compression.

## 1. Introduction

With the rapid development of Internet and computer technology, digital multimedia industry meets huge opportunities as well as great challenges: copyright protection. Utilizing modern techniques, people can easily make the copied digital content identical to the original one. Digital watermarking technology is one of the most effective and efficient techniques to protect copyright under the open network circumstance. Since video products are mostly transmitted in compressed format, it is impossible to design a general watermark scheme applicable to different compression types. Thereby the compression standard must be considered in the design of video watermarking algorithm.

The H.264/AVC standard is the latest video codec released by the Joint Video Team (JVT). As one of the most commonly used formats for the compression and distribution of high definition video, H.264/AVC introduces several new coding methods to improve the rate-distortion performance comparing with the past coding standards, such as MPEG-x and H.263.

The earliest compressed domain watermarking scheme is based on Variable Length Coding (VLC) [1]. Hartung and Girod [2] firstly proposed embedding spread-spectrum watermark into compressed video for content protection. For the payload of watermark, Biswas et al. [3] embedded watermark by modifying Discrete Cosine Transform (DCT) coefficients. Kutter et al. [4] proposed the first video watermarking scheme involving motion vectors and focused on the parity of motion vector. From then on, several schemes based on motion vector were devised with different selection algorithms. Chen et al. [5] followed a common criterion based on the magnitude and the watermark position from the phase angle of a certain motion vector. Feng and Wu [6] proposed selecting motion vectors with small splitting and minimizing the Laragian function in motion search. Aly [7] chose the candidate subset of motion vectors according to their associated macroblock prediction error. Apart from motion vector, block types and modes of intracoded blocks were regarded as payload by Yang et al. [8].

Insofar as evaluation criteria of watermark, imperceptibility is the basic principle, whereas the watermark embedding operation can easily import distortion drift, leading to a severe visual impact. Ma et al. [9] embedded data into the quantized DCT coefficients of I-frames and utilized direction of intraframe prediction to avert distortion drift in intraframe scenario. Zeng et al. [10] adopted drift compensation to prevent interframe error propagation, but this method requires a partial reconstruction of some pixels. Gong and Lu [11] avoided employing drift compensation by attaching watermark embedder with the video encoder, yet the computation burden increased inevitably. Hence, an effective and efficient algorithm to offset distortion drift is of high significance for motion vector based scheme.

Another important concern of watermark is the robustness against unintentional/intentional attacks. Langelaar and Lagendijk [12] proposed the Differential Energy Watermarking (DEW) algorithm for the first time, which could resist reencoding attacks. Wu et al. [13] devised a blind watermarking algorithm with I-frames as payload, which successfully survives H.264 compression attacks with a larger than 40 : 1 ratio in I-frames; however, it requires decompressing the video in order to execute embedding operation. Noorkami and Mersereau [14] also took robustness into consideration, yet the original (uncompressed) video is required for calculating the parameter of visual model, resulting in a computationally expensive prediction process. Therefore, the demands of watermark call for a robust watermarking scheme which can resist attacks and maintain low computational complexity at the same time.

The objective of this paper is to present a robust motion vector based watermarking method for H.264 video stream with drift compensation. DCT transform is adopted to motion vector residual groups in order to generate energy relationship between two certain groups, and this relationship can conserve stability under attacks. Experimental results indicate that the proposed scheme is robust to intentional attacks with no visual distortion influence.

The rest of this paper is organized as follows. In Section 2, some important features of H.264/AVC are introduced.  Section 3 presents our watermarking scheme.  Section 4 demonstrates the experimental results and analysis. Conclusions are drawn in Section 5.

## 2. Relevant Features for H.264/AVC Video Compression

H.264/AVC contains a number of new features that allow it to compress video much more effectively than older standards and to provide more flexibility for application to a wide variety of network environments. Tree structured block size, motion compensation, and distortion drift provide theoretical support for our watermarking scheme.

### 2.1. Tree Structured Block Size

**Figure 1:**
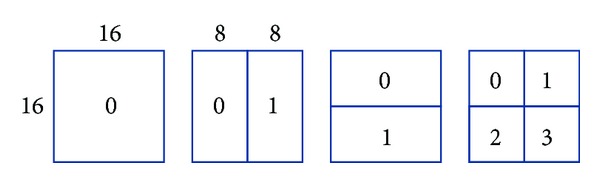
Macroblock partitions: 16 × 16, 8 × 16, 16 × 8, or 8 × 8.

H.264/AVC supports motion compensation block sizes ranging from 16 × 16 to 4 × 4 luminance samples with many options. The luminance component of each MB (macroblock) (16 × 16 samples) may be split up in 4 ways as shown in Figure 1.

**Figure 2:**
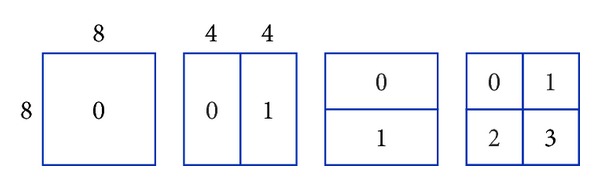
Macroblock subpartitions: 8 × 8, 4 × 8, 8 × 4, or 4 × 4.

Each of the subdivided regions is a MB partition. If the 8 × 8 mode is chosen, each of the four 8 × 8 MB partitions within the MB may be further split in 4 ways as shown in Figure 2.

These partitions and subpartitions give rise to a large number of possible combinations within each MB [15].

### 2.2. Motion Compensation

In block motion compensation, the frames are partitioned into blocks of pixels (e.g., MB of 16 × 16 pixels). Each block is predicted from a block of equal size in the reference frame. The blocks are not transformed in any way apart from being shifted to the position of the predicted block. This shift is represented by a motion vector.

To exploit the redundancy between neighboring block vectors (e.g., for a single moving object covered by multiple blocks), it is common to only encode the difference between current motion vector and previous motion vector in the bitstream. The result of this differencing process is mathematically equivalent to a global motion compensation that is capable of panning. Then, an entropy coder will take advantage of the resulting statistical distribution of the motion vectors around the zero vector to reduce the output size.

### 2.3. Distortion Drift Theory

In motion compensation, there are three kinds of motion vectors named MV, MVP, and MVD. Apparently, MV is the abbreviation of motion vector, which is a two-dimensional vector used for interprediction that provides an offset from the coordinates in the decoded picture to those in a reference picture. MVP means motion vector prediction, which represents the motion vector in the reference picture. MVD stands for motion vector difference, that is, the coded and transmitted motion vector residual. The relationship among these vectors is as follows:

(1) $$\begin{array}{c} \text{MVD}=\text{MV}-\text{MVP}. \end{array}$$

Altering the value of motion vector difference will make the embedded MB change, which means that a certain block in frame becomes different. What is more, this distortion is then propagated to the adjacent MBs and succeeding frames due to motion prediction compensation, even though these MBs and frames are not watermarked at all. This is called distortion drift and methods adopted to prevent such distortion from happening are called drift compensation.

**Figure 3:**
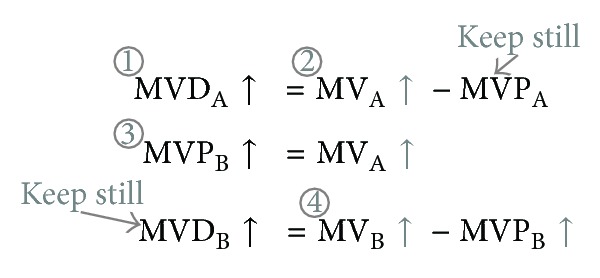
Example of distortion drift.

For instance, we have two 16 × 16 MBs named as A and B, where A is the reference block of B in the process of median prediction which means MVP_B_ = MV_A_. MVD_A_ being increased by 1 (step *①* in Figure 3) leads to MV_A_ (step *②* in Figure 3) as well as MVP_B_ (step *③* in Figure 3) increasing by 1 given that MVP_A_ is not modified. Even with MVD_B_ unchanged, MV_B_ will finally increase by 1 (step *④* in Figure 3). Such influence will spread to MBs in a certain scope and cause severe distortion drift, as can be seen in Table 1.

## 3. Proposed Video Watermarking Scheme

**Figure 4:**
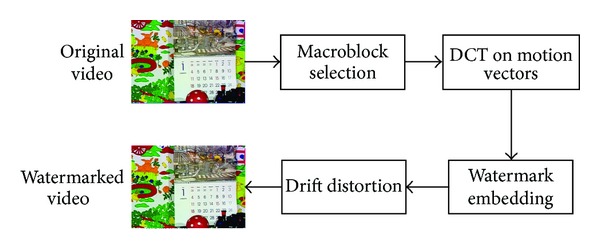
Framework of our scheme.

Our proposed watermarking scheme is shown in Figure 4. First, in consideration of imperceptibility, proper macroblocks should be selected according to the syntax element in H.264 named MB_TYPE to reduce the visual impact of embedded watermarks. Reasonable macroblock selection will also diminish the probability of distortion drift. Second, DCT will be applied to groups of the selected motion vectors to utilize the “energy compact” property so that intentional attacks can be resisted. The watermarks are embedded in the DC and 2 AC coefficients mostly adjacent to DC coefficient. Finally, drift compensation is implemented to avoid distortion drift.

### 3.1. Watermark Generation

In our experimental works, one binary image is taken as the content of the watermark. To improve the security of our algorithm and the robustness of embedded watermark, pseudorandom permutation with a secret key is performed on the watermark image before embedding.

Assume that *T* ^2^ is the original watermark image with binary value. Use the given secret key *K* to perform Arnold transform on *T* ^2^. Regarding *T* ^2^ as the 2-dimensional image space, the Arnold transform is the transformation Γ : *T* ^2^ → *T* ^2^ given by

(2) Γ([xy])=[2111][xy]mod⁡K,

where *K* is the chosen secret key and pixel value at (*x*, *y*) is replaced by value at (*x*′, *y*′) calculated from the above formula.

With such a transformation, a scrambled image can be generated through pixel permutation. If secret key is unavailable, no readable information can be obtained even after watermark being correctly detected.

**Figure 5:**
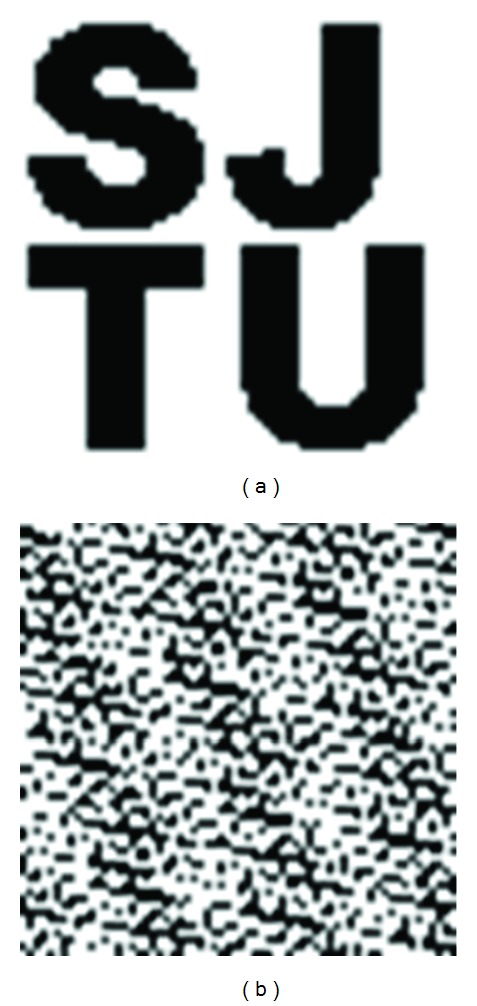
Images before and after Arnold transform.

Finally, the binary image is converted into a scrambled binary sequence. This scrambled sequence is the watermark signal to be embedded; see Figure 5. This process can be denoted as

(3) W=B(Γ(T2,K)),

where Γ is Arnold's transform, *B* is the process to generate binary sequence, and *W* is the watermark signal to be embedded.

### 3.2. Macroblock Selection

As indicated in Table 2, to maintain the imperceptibility of watermark, those MBs with “mb_type” = 4 are selected to embed watermark in our scheme, because such MB contains four 8 × 8 sub-MBs whereas “mb_type” = 0, 1, 2 means one 16 × 16 MB, two 16 × 8 MBs, and two 8 × 16 MBs, respectively. The P_8 × 8ref0 prediction mode is just a short-hand way of expressing a combined selection of P_8 × 8 and ref_idx_l0 = 0 which is just a requirement for the bitstream. Furthermore, these 8 × 8 sub-MBs may be split into more subpartitions, which helps to reduce the visual influence to the most extent. In our scheme, we select the right bottom sub-MB and the right bottom subpartition is the final place to embed watermark when subpartition exists.

Actually, we have another reason to choose the right bottom sub-MB/subpartition to embed watermark. As mentioned above, changes in one MB may cause distortion drift once this MB is referred by other MBs. But the right bottom sub-MB/subpartition is the least possible one to be referred by adjacent MBs.

**Figure 6:**
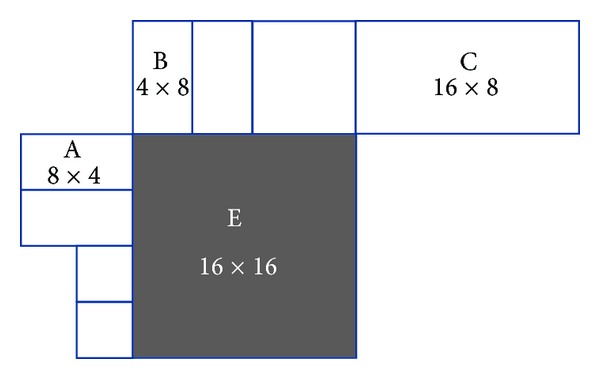
Current block and adjacent blocks with subpartitions.

Specifically, in the motion vector median prediction, suppose E in Figure 6 is the current MB/sub-MB/subpartition, A is on the left side, B is on the top, and C is on the up right side. If more than one block is on the left side of E, then choose the uppermost as A. The leftmost one as B may be chosen under the same consideration. As you can see, with a right bottom 8 × 8 sub-MB or even 4 × 4 subpartition selected to embedded watermark, the possibility to be taken as reference MB is the lowest.

### 3.3. Watermarking Embedding Scheme

**Figure 7:**
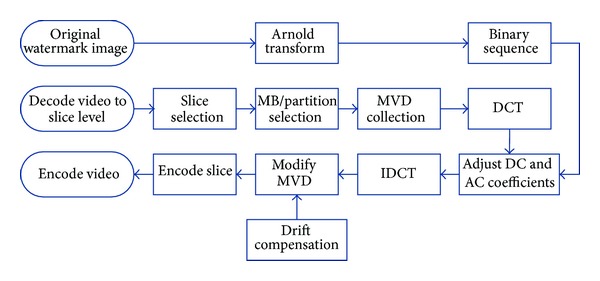
Watermark embedding approach.

Figure 7 illustrates our watermark embedding approach. The detailed embedding process is as follows.

**Figure 8:**
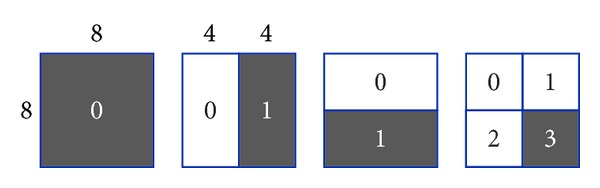
Watermarked macroblock subpartitions: 8 × 8, 4 × 8, 8 × 4, and 4 × 4.

(1) The target video should be decoded until reaching the slice level.

(2) When encountering I/B slice, skip to the next slice. Only if the current slice is P slice, proceed further decoding.

(3) Decode MB syntax of P slice. Only the P MBs containing four 8 × 8 sub-MBs are selected. Find the right bottom sub-MB (8 × 8) and read motion vectors of the right bottom subpartition if subpartition exists. In Figure 8, the horizontal motion vector residuals of shadowed partition are used to embed watermark.

(4) Take a line as a unit and start searching from the right bottom corner of the slice. If we succeed to collect 8 P MBs, continue to search the next line until we gather 32 MBs (4 lines are needed):

(4) MVD∈⋃j=0 3⋃i=07 MVDij, if  mb_type=4,

where the expression on the right-hand side represents the motion vector residual group which meets the requirement.

(5) Divide these 32 MBs into two groups (16 MBs each), and then process horizontal MVDs of the right-bottom subpartition in each MB by modulus 10; that is, extract units digit of each MVD:

(5) MVD′=⋃j=0 1⋃i=015MVDijmod⁡10,

where the expression on the right-hand side means the two divided motion vector residual groups each containing 16 elements. The MOD 10 operation aims to reduce the energy difference between two groups, leading to a relatively small gap. Thus, if we need to reverse the energy relationship between two groups in order to embed watermark, only a slight modification of DCT coefficients is enough, which will in turn only affect the units digits of motion vectors.

(6) Convert each group into 4 × 4 matrix and perform DCT transform:

(6) MVDk1k2′′=∑n2=0 3∑n1=03MVDn1n2′cos⁡[π3(n1+12)k1]×cos⁡[π3(n2+12)k2]k1=0,…,3,  k2=0,…,3.

(7) According to the watermark bit to be embedded, modify the value of DC coefficients and 2 low-frequency AC coefficients which are mostly adjacent to DC coefficients according to

(7) MVDA′′−MVDB′′>T, if  watermark=1,MVDA′′−MVDB′′≤T, if  watermark=0,

where MVD_A_′′ and MVD_B_′′ are the sum of DC and 2 AC coefficients from group A and group B, respectively. *T* is the threshold which is set to 5 according to experimental results.

(8) Take IDCT transform, and then calculate the final motion vectors as follows:

(8) MVD¯¯k=MVD−MVD mod 10+MVD¯k, k=0,…,31,

where ${\bar{\text{MVD}}}_{k}$ is the IDCT result after DCT coefficients modified, and MVD is the original horizontal motion vector residual. Therefore, the final motion vector ${\bar{\bar{\text{MVD}}}}_{k}$ has the same tens digit with the original one, only with minor change in units digit.

(9) Encode the current P slice.

(10) Loop from step (2) until we reach the end of slice.

Comparing with previous watermarking schemes based on motion vector, our scheme can survive intentional attacks such as lossy compression or motion estimation algorithm change. Because for traditional watermarking schemes which regard the parity of motion vector as payload, the only one bit difference in parity will vanish after being reencoded, the embedded watermark cannot survive after some normal operations, let alone intentional attacks.

We adopt DCT in our scheme for its “energy compaction” property [16]: most of the signal information tends to be concentrated in a few low-frequency components of the DCT. Specifically, when confronted with some unintentional/intentional attacks, value of motion vector residuals will undoubtedly change, but the gross energy of motion vectors in a certain group will not change a lot so the energy relationship between two groups will be quite stable even with lossy compression operation. That is the reason that this design in our scheme is robust to intentional attacks.

### 3.4. Watermarking Detection Scheme

**Figure 9:**
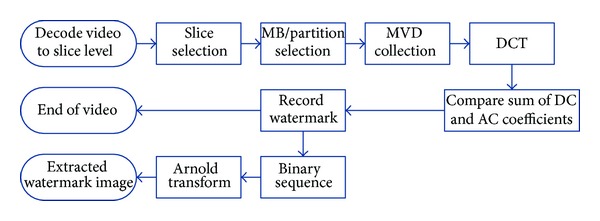
Watermark detection approach.

Figure 9 demonstrates the watermark detection approach. The watermark detection scheme is described as follows.

(1) The watermarked video is decoded until slice level is reached.

(2) Only decode the current slice when it is P slice and skip to the next slice for I/B slice.

(3) Decode MB syntax of P slice. Search for P MBs containing 8 × 8 sub-MBs. Read motion vectors of each sub-MB and record horizontal MVD of the right bottom sub-MB.

(4) Take a line as a unit and start searching from the right bottom corner. If we succeed to collect 8 P MBs, continue to search the next line until we get 32 MBs (4 lines in all).

(5) Divide these 32 MBs into two groups (16 MBs each), and then process horizontal MVDs of the right bottom subpartition in each MB by modulus 10.

(6) Convert each group into 4 × 4 matrix and take DCT transform.

(7) Make comparison between the sum of DC and the 2 nearest AC coefficients in two groups:

(9) $$\begin{array}{c} \text{watermark}=1,\;\text{if}\;\text{MV}{\text{D}}_{\text{A}}^{\prime \prime }-\text{MV}{\text{D}}_{\text{B}}^{\prime \prime }>T,\\ \text{watermark}=0,\;\text{if}\;\text{MV}{\text{D}}_{\text{A}}^{\prime \prime }-\text{MV}{\text{D}}_{\text{B}}^{\prime \prime }\leq T. \end{array}$$

(8) Loop from step (2) until we reach the end of slice.

### 3.5. The Mechanism of Drift Compensation

For the example mentioned in Section 2.3, we can easily make minus 1 to compensate the increase due to watermarking.

**Figure 10:**
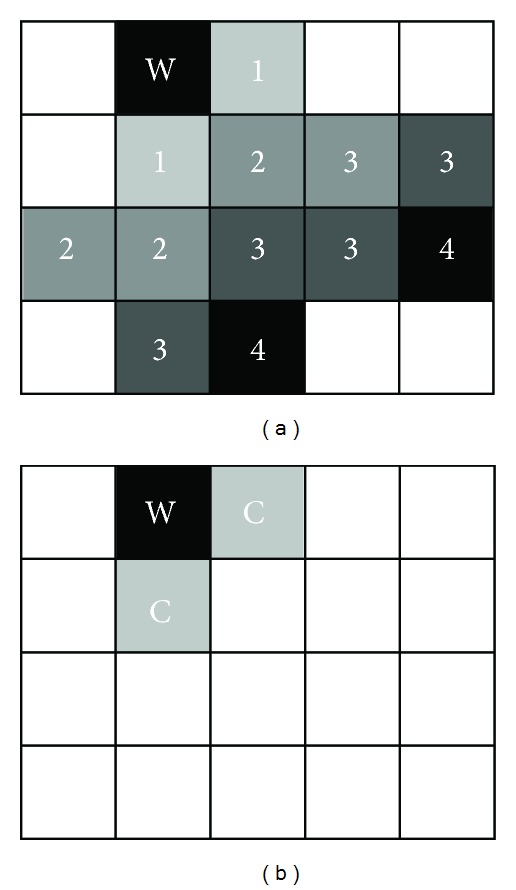
Distortion drift and drift compensation.

As shown in Figure 10(a), the black block “W” indicated the block being watermarked and all grey blocks are influenced by distortion drift. The number in block means the order of impact. “1” blocks are impacted by “W” blocks and “2” blocks are changed because of “1” blocks. For Figure 10(b), “C” blocks are the blocks being compensated so that no further impact happens on the adjacent blocks.

Since general circumstances are more complicated, the following steps should be taken to carry out drift compensation.

**Step 1**

Store information (MB number and amount of modification) of previous MB which has MVD changed because of watermark embedding.

**Step 2**

For the case that the reference MB of current MB has been watermarked according to the information recorded in Step 1, consider the following.

(a) If the current MB is not selected to embed watermark, just perform reverse modification according to the reference MB.

(b) If the current MB is selected to embed watermark, do the embedding modification and store information of current MB. However, the two modifications on current MB and the reference MB need to be accumulated; hence, drift compensations can be conducted together next time.

However, even though it is possible to know which MB is taken as reference in the process of decoding, the reference MB will sometimes change to other MB when modification is performed. Thus the drift compensation may cause worse effects. The reason for such change in reference MB is that in the interprediction stage, three adjacent MBs are taken into consideration and MB with median motion vector value is selected as reference one. Modification on MVD will change MV so that this increase/decrease in motion vector value will impact the relationship of the three MBs and lead to the alteration of reference MB.

To solve such a problem, more work needs to be done before Step 2.

- If median prediction is disabled for the current MB, the reference MB before and after embedding will not change. Just go to Step 2.
- If median prediction is chosen, retrieve MVs of three adjacent MBs and increase or decrease them according to the modification information stored. Thus through calculating actual median value, the reference MB after embedding can be determined.
- If reference MB stays the same, go to Step 2.
- If change happens, denote new modification as the difference between MV of new reference MB and MV of the original reference MB. Then go to Step 2. Specifically, for the case where reference MB changed, different MVPs will render MV of this MB erroneous. Hence, by adding/subtracting new modification amount, final MV of this MB can be rectified to the original value before being watermarked.

**Figure 11:**
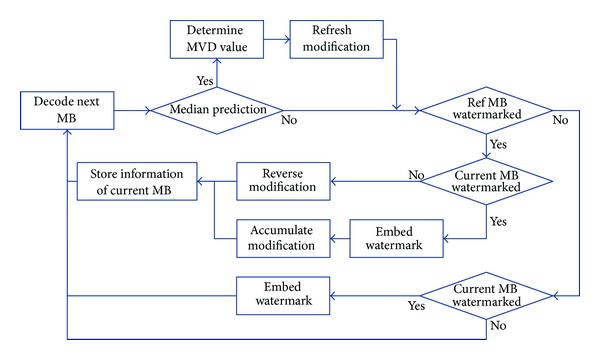
Drift compensation approach.

The drift compensation approach is illustrated in Figure 11.

## 4. Experimental Results

In this experiment, the H.264/AVC codec JM8.6 [15], officially released by the Joint Video Team (JVT), is used to test universal standard test sequence (CIF), 352 × 288 “Bus,” “Flower,” “Mobile,” and “Stefan,” respectively. All of them use context-adaptive binary arithmetic coding (CABAC) method with a frame rate of 20 frames per second.  Table 3 shows the experimental parameters.

### 4.1. Watermark Imperceptibility

According to the experimental conditions mentioned, we set the default quantization parameter (QP) to 28, choose IPPP type as coding sequence, and select “Bus,” “Flower,” “Mobile,” and “Stefan” sequences for testing. A video quality analysis software named “Elecard Stream Tools” is employed in our experiment.

#### 4.1.1. Comparison with Original Video

Table 4 shows one frame of the reconstructed image before and after the four decoded video streams being watermarked. The bright area in “Difference” row means the difference between the original video and the watermarked video. As we can see from the above table, the embedded watermark does not affect the subjective video quality of the reconstructed image.

To evaluate the change of video quality before and after being watermark embedded, we introduce four new objective evaluation indicators:

(i) NQI: new quality metrics;

(ii) VQM: Video Quality Measurement techniques;

(iii) SSIM: structural similarity;

(iv) MOVIE: motion-based video integrity evaluation [17].

These four indicators can present numerical difference between the original and watermarked video. With the use of Elecard Video Quality Estimator, we encoded video sequences with different QP values. Results are shown in Table 5.

NQI takes the brightness distortion, contrast distortion, and relativity loss into account, ranging from −1 to 1. NQI closer to 1 means a higher video fidelity. As indicated from Table 5, NQI values range from 0.9678 to 0.9966, and most of these values are above 0.97, which means that our scheme has little influence on video perceptibility. With the increase of QP value, NQI has a slight tendency to drop down, because QP is also a significant factor which affects the video quality to some extent.

VQM is used to measure perceptual effects of video impairments including blurring, unnatural motion, global noise, block distortion, and color distortion. VQM closer to 0 means a smaller distortion and it is sensitive to QP value. As shown in Table 5, VQM values range from 0.2096 to 0.6908 and are mostly smaller than 0.5, which means a minor variance between original and watermarked images. Therefore, the capability to stabilize VQM under 0.5 implies good imperceptibility of our watermarking scheme.

SSIM is a new indicator measuring the similarity of two video frames whose range is [0, 1]. It is based on measuring three components (luminance similarity, contrast similarity, and structural similarity) and combining them into result value. SSIM closer to 1 indicates a higher similarity of two videos. From data in Table 5, maximum value is 0.9958, minimum value is 0.9822, and most of the values are above 0.98, which can illustrate that there is almost no impact on video similarity before and after being watermark embedded.

MOVIE is a general, spatiospectrally localized multiscale framework for evaluating dynamic video fidelity that integrates both spatial and temporal (and spatiotemporal) aspects of distortion assessment. Better video fidelity is obtained when value of MOVIE is closer to 0. As shown above, the MOVIE ranges from 0.000890 to 0.005121. So we can conclude that the video fidelity has been little influenced after being watermarked.

#### 4.1.2. Experiments on Drift Compensation

**Figure 12:**
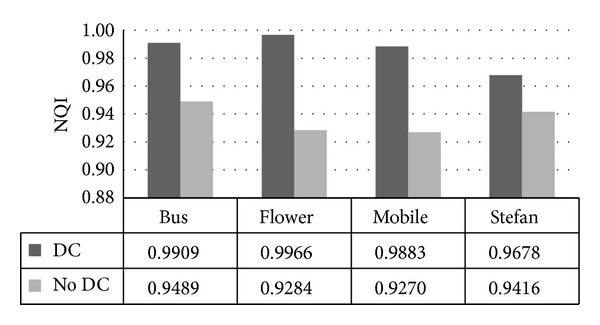
NQI comparison between schemes with DC and without DC (DC stands for drift compensation).

**Figure 13:**
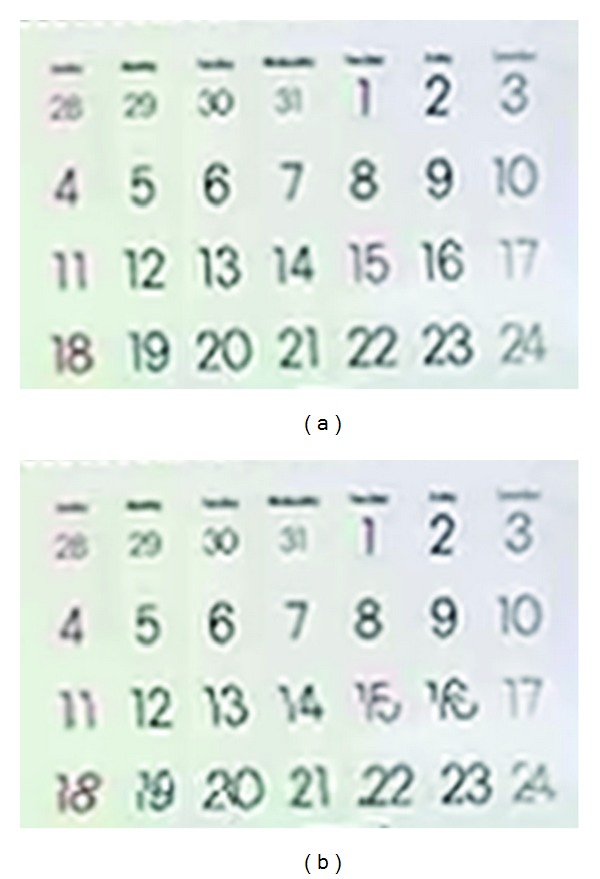
Effect of decrease on NQI by 0.0613.

Drift compensation is adopted in our scheme to avoid distortion drift and to improve the quality of watermarked video. As mentioned before, NQI closer to 1 indicates a better imperceptibility. As shown in Figure 12, scheme with drift compensation (DC) obtains a higher NQI value compared with scheme without drift compensation for all four test samples. The difference reaches 0.0682 at most, and Figure 13 shows results for “Mobile” whose NQI drops to 0.0613. Conclusion can be drawn that drift compensation has significant effect on reducing the distortion caused by watermarking.

**Figure 14:**
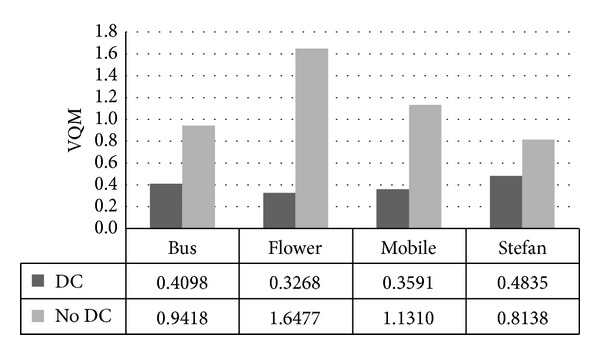
VQM comparison between schemes with DC and without DC.

Lower VQM value indicates better video quality and Figure 14 shows that scheme using drift compensation gains much lower VQM values and this value increases to 1.3209 at most than scheme without it. Therefore, drift compensation can ensure better imperceptibility.

**Figure 15:**
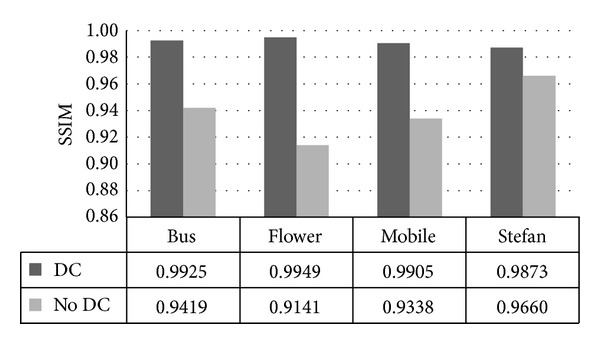
SSIM comparison between schemes with DC and without DC.

**Figure 16:**
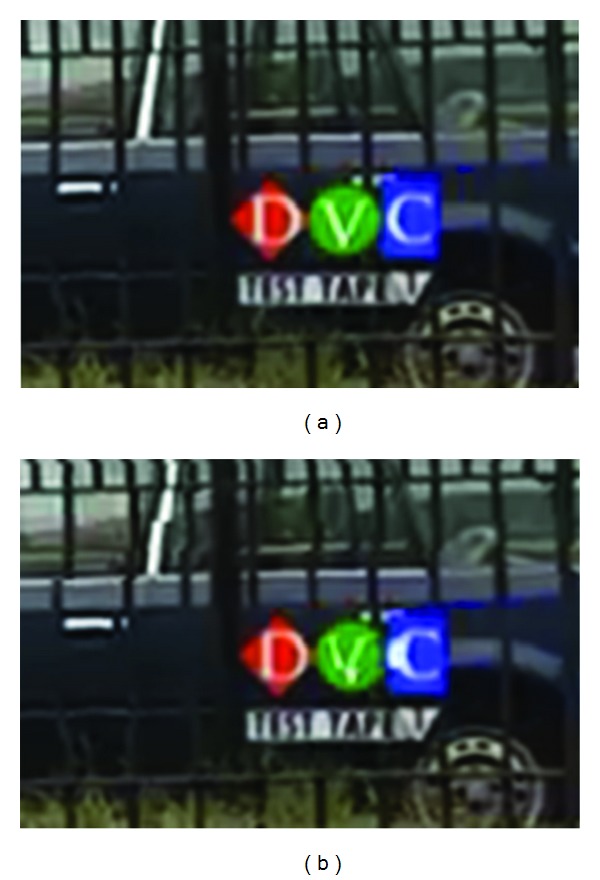
Effect of decrease on SSIM by 0.0506.

We find that SSIM value drops fiercely if drift compensation is not carried out in our scheme as shown in Figure 15. The drop of SSIM value is 0.0808 at most, which means a great decrease in video quality.  Figure 16 shows results for “Bus” whose NQI drops to 0.0506. Hence, scheme using drift compensation gains a better imperceptibility.

**Figure 17:**
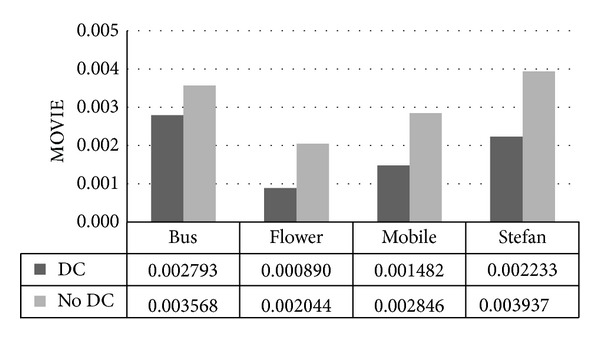
MOVIE comparison between schemes with DC and without DC.

As indicated above, lower MOVIE is obtained when drift compensation scheme is applied. The largest difference in Figure 17 is 0.001704 for “Stefan,” whereas the smallest difference is 0.000775 for “Bus.” The import of drift compensation leads to a 40% drop in MOVIE on average. So we can conclude that imperceptibility of scheme with drift compensation is better than that without it.

#### 4.1.3. Comparison with Other H.264/AVC Schemes

To analyze the imperceptibility of our scheme, we compare our scheme with Sun et al.'s algorithm [18].

**Figure 18:**
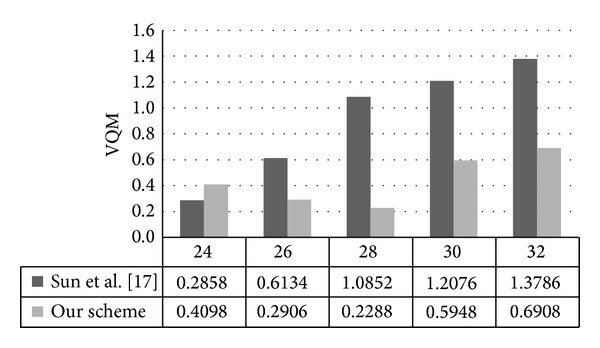
Comparison of imperceptibility performance.

In this experiment, we use sequence “Bus” with QP ranging from 24 to 32. As shown in Figure 18, when QP = 24, the VQM value of our scheme is slightly larger than Sun et al.'s scheme [18], but for the case of QP = 26,28,30,32, our VQM values are much smaller than Sun et al.'s scheme and show a better stability. The reason is that Sun et al.'s scheme embedded watermarks in VLC domain so that it performs well in high bit-rate condition, whereas the influence of watermark will become inevitable when confronted with quality decrease situation.

Conclusions can be drawn from all of the data above.

(1) For most of the time, NQI is above 0.97, VQM is below 0.5, SSIM is above 0.98, and MOVIE is below 0.005. All these four indicators demonstrate that our scheme has excellent imperceptibility.

(2) For scheme without drift compensation, NQI drops to 0.06, VQM increases to 1.3, SSIM decreases to 0.08, and MOVIE increases to 0.001704 at most. Our scheme with drift compensation gains better video quality.

(3) In comparison with Sun et al.'s [18] scheme, better performance can be obtained in our scheme when QP ranges from 26 to 32.

### 4.2. Robustness against Intentional Attacks

In this experiment, all four sequences “Bus,” “Flower,” “Mobile,” and “Stefan” are used. After watermark is being embedded, these videos are decoded and reencoded with different QP values and motion estimation algorithms to generate recompression.

#### 4.2.1. Intentional Attack with Different QP Values

To verify the robustness under lossy compression, the watermarked video is recompressed with different QP values.  Table 6 illustrates the effect on content after compression. The watermark images detected from each recompressed video are shown in Table 7.

In Table 6, the bright areas indicate differences with the original frame. The impact of QP value on video quality is visually obvious. As illustrated by Table 7, the watermark image changes more or less after attack. With the increase of QP value, the compression rate becomes higher and the loss of watermark information becomes severer. However, the pattern can still be easily recognized, and the lowest correct rate is still above 70%, which is defined as follows:

(10) $$\begin{array}{c} \text{Acc}=\frac{c}{w}, \end{array}$$

where Acc is correct rate, *w* is the bit number of a certain watermark, and *c* is the number of bits correctly detected.

**Figure 19:**
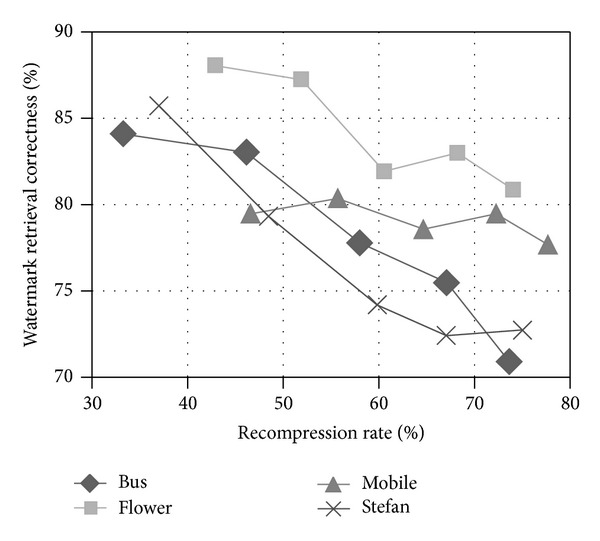
Watermark correctness rate after lossy compression with different QP values.

Figure 19 shows the robustness of our watermark scheme against lossy compression attack with different QP values. The recompression rate defined in (11) ranges from 40% to 80% as

(11) $$\begin{array}{c} R=1-\frac{S_{w}}{S_{o}}, \end{array}$$

where *R* stands for the recompression rate, *S*_*w*_ and *S*_*o*_ denote watermarked video size and original video size, respectively.

As can be seen from Figure 19, under a lossy compression rate less than 50%, the correctness rate can keep above 79%, and the best correctness can be as high as 88% with the lowest recompression rate 40%. Even with a high recompression rate of 80%, the correctness of watermark detection can still reach 70%. The correctness rate decreases as the lossy compression rate increases and such decline is much more apparent for sequences “Bus” and “Stefan,” because the motion amplitude of objects in these two videos is quite huge and the lossy compression will have a significant impact on the motion estimation with relatively large original motion vectors.

#### 4.2.2. Intentional Attack with Different Motion Estimation Algorithms

Motion estimation algorithm is the most relevant factor for motion vector because different motion estimation algorithms will cause huge change to the final motion vector.

In this experiment, we keep the QP value unchanged and choose different motion estimation algorithms in the process of encoding. We firstly use JM8.6 to generate video files with embedded watermark and decode video files into YUV sequences. A video encoder software named X264 is then used to encode these YUV sequences into video files again. However, different motion estimation algorithms are chosen in this encoding process so that intentional attack can be performed.

The default motion estimation algorithm of JM8.6 is “Fast Full Pel Block Motion Search,” whereas X264 has five different motion estimation algorithms named “Diamond Search,” “Hexagonal Search,” “Uneven Multi-Hexagon Search,” “Exhaustive Search”, and “Transformed Exhaustive Search.”

**Figure 20:**
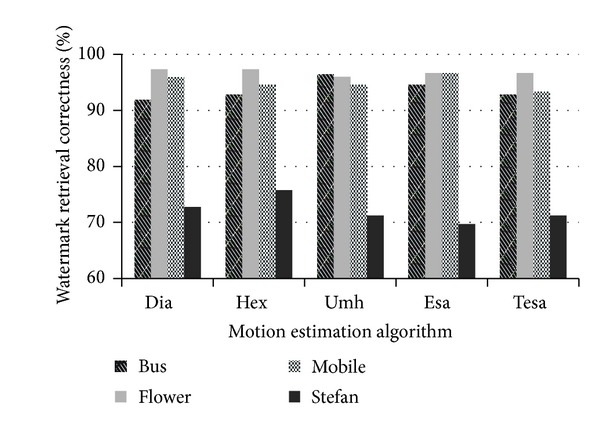
Watermark correctness rate after recoding with different motion estimation algorithms.

As indicated in Figure 20, the robustness of our watermark scheme against reencoding with different motion estimation algorithms is satisfactory. Under most circumstances, the correctness rate is above 90%. The results of “Stefan” are relatively below normal level because this video sequence contains fierce movements so that the large motion amplitude will be greatly influenced by the chosen algorithm.

As mentioned before, few research works focused on robust watermarking scheme based on motion vector, so it becomes extremely difficult to do comparison experiments with other proposed algorithms. The most relevant literature we found is proposed by Li et al. [19] which focused on MPEG-2, but it is still quite different compared with our scheme on H.264/AVC.

In our experiment, an extreme lossy compression rate 80% is tested and such high compression rate will cause great decline in video quality. Nevertheless, the correctness of watermark detection can still keep above 70% under such a circumstance. In the meantime, the watermark retrieval correctness has a high stability under different motion estimation algorithms. Conclusion can be drawn that our scheme has an excellent robustness against intentional attacks.

### 4.3. Watermark Capacity Analysis

The capacity of a watermarking scheme decides the application scope of the algorithm. With our proposed scheme, different bits of watermark are embedded into video and visual impact is observed carefully. As a result, two-bit watermark can be embedded into each P frame and little visual decrease can be noticed. But if we try to embed 3 bits in each frame, the imperceptibility of our scheme will suffer a slight decrease occasionally.

As illustrated in Table 8, with two bits embedded, the video quality has no visible decrease compared with the original video frame, whereas given that 3 bits are embedded in one frame, some distortion is visually obvious. For this reason, we conclude that at most two bits can be embedded into one frame. For an “IPPP” GOP type, each of the four frames has 6 bits embedded, which means that an average capacity of 1.5 bits per frame can be achieved.

Videos are usually displayed at a rate of more than 24 frames per second so that at least 22.5 bits of covert information can be embedded within one second in video. With content of watermark elaborately designed, we may embed one entire watermark within several seconds (video time).

### 4.4. Impact on Video Bit Rate

Bit rate is an important feature of video quality, especially for those low bit rate H.264 videos which have a high request on rate control. The watermark embedding is bound to affect the video bit rate. The ability to control bit rate effectively and strictly is also an important indicator of H.264 video watermarking scheme.

**Figure 21:**
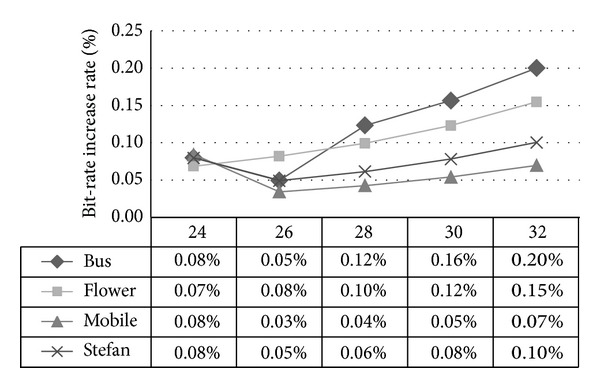
Bit-rate increase rate for different sequences and QPs.

Figure 21 shows the comparison of bit-rate change of all four sequences encoded with different QP values, from which conclusion can be drawn that the bit-rate increase rate ranges from 0.03% to 0.20%. The average of bit-rate increase rates is 0.09%, which is acceptable for normal watermarking scheme. According to the data analysis, the bit-rate of each video slightly increases along with the increase of QP value, but no sudden change has occurred and the range of increase is limited. For normal circumstance, QP = 28 is mostly used and 0.12% is the biggest increase rate when QP = 28. So the scheme can effectively control the video rate change before and after being watermark embedded.

## 5. Conclusion

In this paper, a robust video watermarking scheme with drift compression is proposed. The devised MB selection scheme can lower the influence on video quality and reduce the possibility of drift distortion. In the meantime, a new drift compensation scheme is adopted to improve the imperceptibility of watermarking. Besides, the “energy compaction” property of DCT is utilized to embed the watermark into motion vector residual with good robustness against intentional attack. The experimental results indicate that the video quality is almost the same as that of the original one, and the increase of bit rate is less than 0.2%, which keeps the high video compression rate of H.264. This scheme is also robust against malicious attack with different QPs or motion estimation algorithms. Even with a high recompression rate of 80%, the correctness of watermark detection can still reach 70%.

Our future work will focus on the robustness against other video attacks and improving the performance of video sequences containing fierce movements.

## Figures and Tables

**Table 1:** Comparison between original frame and frame with distortion drift.

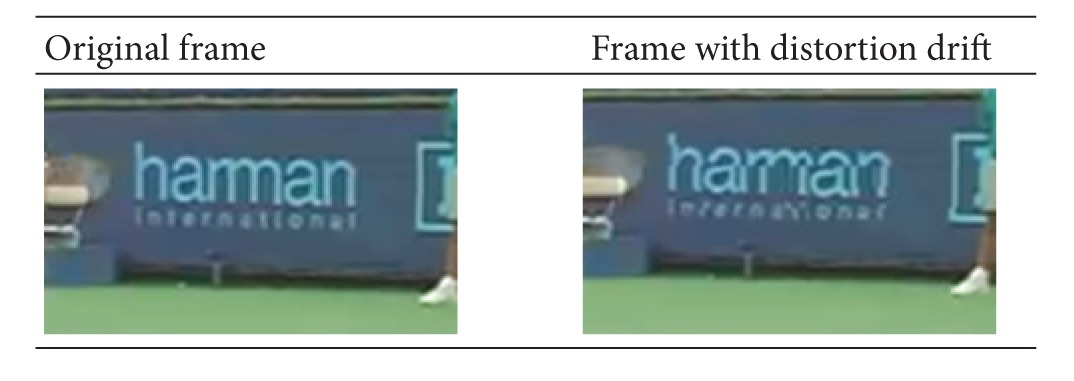

**Table 2:** mb_type in P slice.

mb_type	Type name	Partition amount	Partition width	Partition height
0	P_L0_16 × 16	1	16	16
1	P_L0_L0_16 × 8	2	16	8
2	P_L0_L0_8 × 16	2	8	16
3	P_8 × 8	4	8	8
4	P_8 × 8ref0	4	8	8
N/A	P_Skip	1	16	16

**Table 3:** Parameters of experimental environment.

Profile	Type name
Image size	CIF (352 × 288)
Frame rate	20
Sequence rype	IPPP
Motion estimation	Full search
YUV	4 : 2 : 0

**Table 4:** Comparison between the original video and the video with watermark.

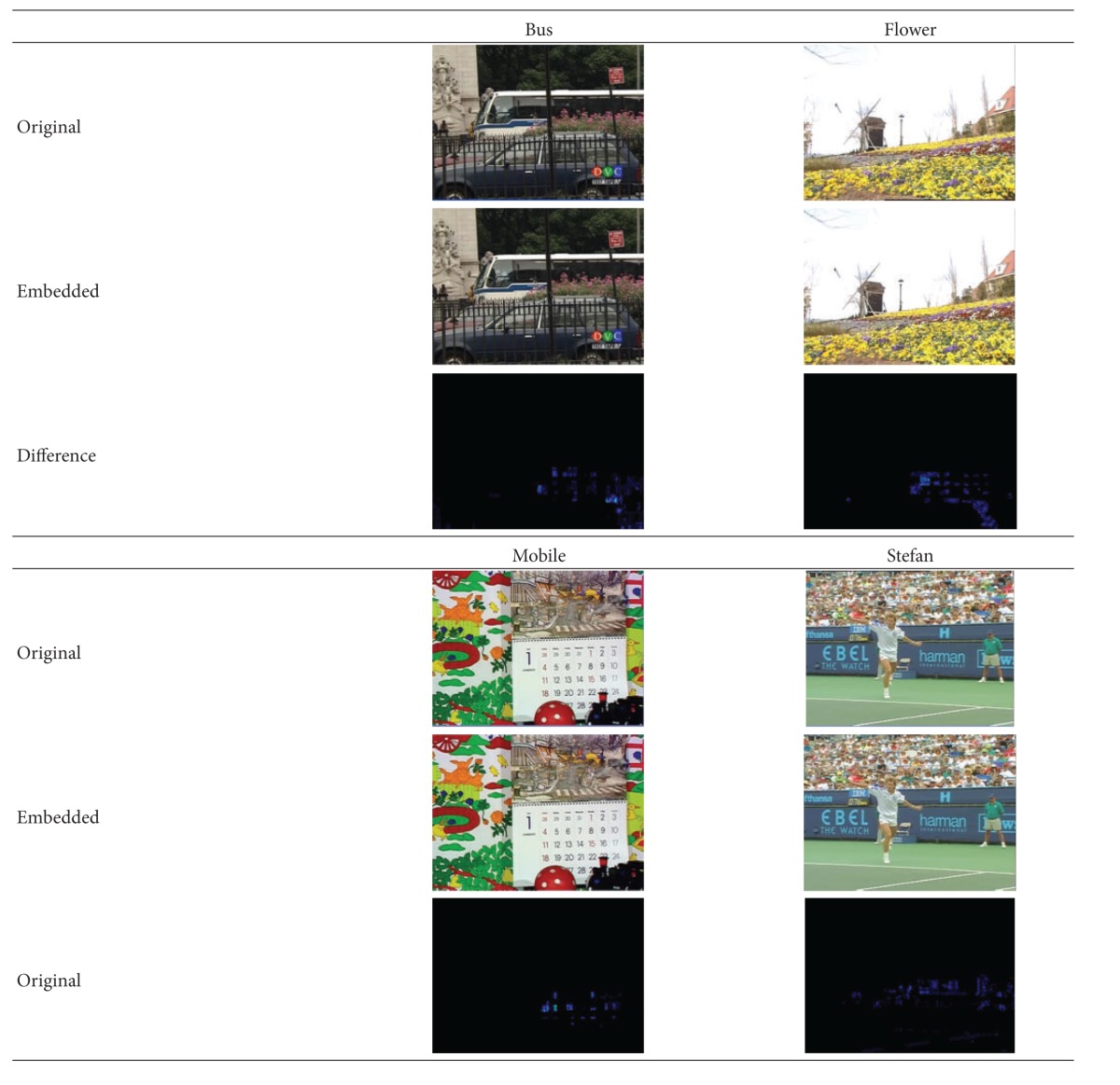

**Table 5:** Results of video quality test.

NQI				
QP	Bus	Flower	Mobile	Stefan
24	0.9909	0.9966	0.9883	0.9678
26	0.9909	0.9956	0.9879	0.9794
28	0.9942	0.9943	0.9895	0.9748
30	0.9833	0.9949	0.9889	0.9730
32	0.9831	0.9956	0.9883	0.9788

VQM				
QP	Bus	Flower	Mobile	Stefan

24	0.4098	0.3268	0.3591	0.4835
26	0.2906	0.3949	0.3520	0.2827
28	0.2288	0.3828	0.2916	0.4275
30	0.5948	0.3655	0.3268	0.3472
32	0.6908	0.3464	0.4459	0.3157

SSIM				
QP	Bus	Flower	Mobile	Stefan

24	0.9925	0.9949	0.9905	0.9873
26	0.9918	0.9934	0.9911	0.9914
28	0.9958	0.9912	0.9913	0.9907
30	0.9833	0.9921	0.9926	0.9861
32	0.9822	0.9931	0.9898	0.9889

MOVIE				
QP	Bus	Flower	Mobile	Stefan

24	0.002793	0.000890	0.001482	0.002233
26	0.003583	0.002265	0.004852	0.002740
28	0.004565	0.002644	0.003575	0.002550
30	0.005121	0.003308	0.004325	0.001656
32	0.004593	0.002943	0.004390	0.002884

**Table 6:** Frame difference with different compression rates.

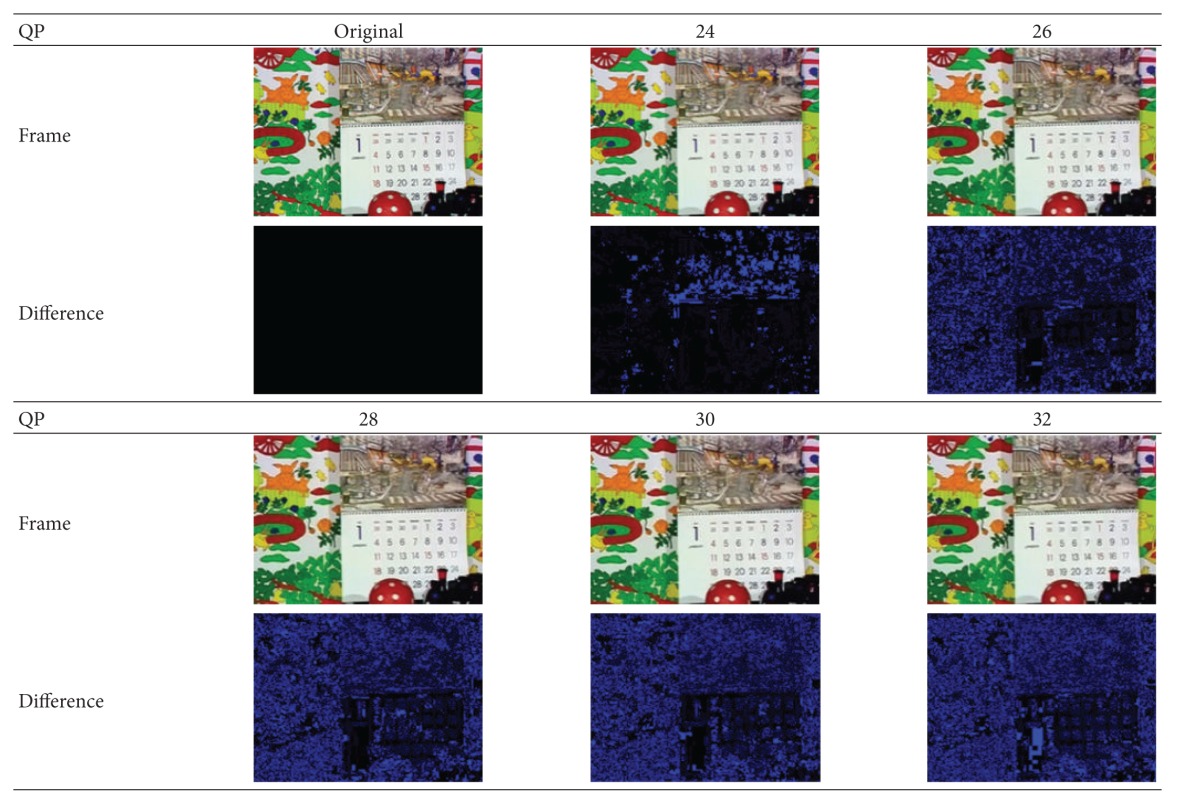

**Table 7:** Watermark images detected from “Flower” after lossy compression attack.

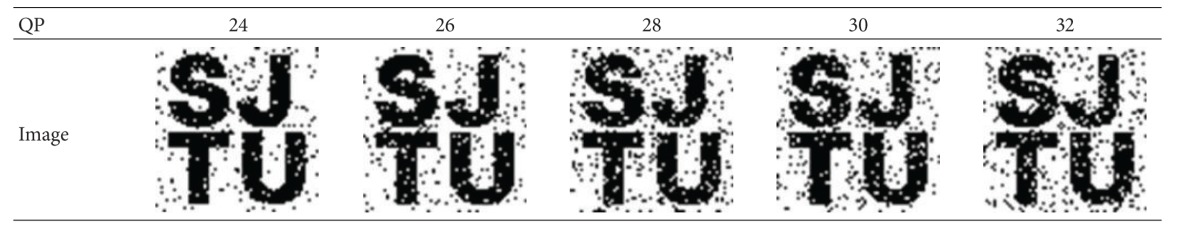

**Table 8:** Comparison of different watermark capacities.